# Feasibility issues impacting optimal levels of maternity care in rural communities: implementing the Rural Birth Index in British Columbia

**DOI:** 10.1186/s12913-022-09008-9

**Published:** 2023-01-04

**Authors:** Jude Kornelsen, Glenys Webster, Stephanie Lin, Nicky Cairncross, Erin Lindstrom, Stefan Grzybowski

**Affiliations:** 1 Centre for Rural Health Research, Department of Family Practice, University of British Columbia, Vancouver, BC Canada; 2 Office of the Representative of Children and Youth of British Columbia (RCY BC), Victoria, BC Canada; 3 Food & Nutritional Sciences, University of British Columbia, Vancouver, BC Canada; 4 Advocate for Service Quality, Government of British Columbia, Victoria, BC Canada; 5 Women’s & Maternal Health, Public Health Prevention and Planning Branch, Population and Public Health Division, Ministry of Health, Victoria, BC Canada

**Keywords:** Rural maternity care, Health service planning, Population need

## Abstract

**Introduction:**

The continued attrition of maternity services across rural communities in high resource countries demands a rigorous, systematic approach to determining population level need, including a clear understanding of feasibility issues that may constrain achieving and sustaining recommended levels of services. The Rural Birth Index (RBI) proposes a robust and objective methodology to determine such need along with attention to the feasibility of implementation.

**Background:**

Predictions of appropriate levels of maternity care in rural communities require consideration of the feasibility of implementation. Although previous work has focused on essential considerations that impact feasibility, there is little research documenting the barriers to implementation from the perspective of rural care providers and administrators.

**Methods:**

We conducted in-depth, qualitative research interviews with rural community health care administrators and providers (*n* = 14) to understand the challenges of offering maternity care in 10 rural communities across British Columbia (BC).

**Results:**

Participants articulated three thematic challenges to providing maternity services in their communities: maintaining clinical skills and financial stability in the context of low procedural volume, recruitment and retention of care providers and challenges with patient transport.

**Conclusions:**

Current models of compensation for maternity care are inadequate and inflexible and underscore many of the challenges to implementing a level of care that is based on population need. Re-thinking provision of care as a social obligation to actualize our system commitment to equity instead of working to achieve economies of scale is the first step to use equitable care. Addressing remuneration will provide the groundwork for solving other barriers to sustainable care.

## Introduction

The enduring challenge of maintaining maternity services across rural Canada, and internationally, requires an approach to planning that assesses population level need, determines the feasibility of implementing the required level of service, provides evidence for system interventions, and iteratively evaluates progress. The Rural Birth Index (RBI) is a tool that helps determine the need for maternity services at a local rural hospital, based on the birth rate within a population catchment around that hospital [1]. It was developed through extensive qualitative research in rural BC communities with varying levels of care, including several that had lost maternity services. It considers the birth rate in communities (averaged over 5 years to account for anomalies), a measure of social vulnerability underscored by the recognition that communities with higher levels of social vulnerability likely require more care, and the travel distance to the next caesarean section service. In the initial calculation, the RBI had a predictive value of accurately representing the appropriate level of care in 33 of 42 communities (79%) [1]. When taken together, these metrics provide a guidepost for health system planners and allows them insight to levels of care relative to communities of like size and characteristics.

Understanding evidence-based service delivery levels is essential to maintaining optimal maternal-newborn health and ensuring service stability. Decades of evidence from Canada, Australia and elsewhere document the consequences of underserviced communities including increased need for birthing families to travel to access care and the attendant consequences (namely financial stress and the anxiety that arises from social displacement) [2–4]. This has been documented to be more acutely felt in some Indigenous communities [5, 6]. There is also mounting evidence of increased adverse outcomes for families that need to travel more than one hour to access care [7, 8]. However, *over* serviced communities also suffer consequences of lack of appropriate levels of care including increased intervention rates, challenges retaining providers and undermining the smaller surrounding services [1]. That is, maintaining enough volume to support specialists call groups may incent lack of outreach support in favor of centralizing care in larger centres. Beyond the health system implications, mal-distribution of health care resources violates the fundamental principles of equity, accessibility and efficiency, and moves us away from the values proposition of proportionate universalism: that is, allocating resources to those who need them the most.

Accurately assessing population-level need is an essential first step in effective maternity services planning. However, deterministic (often top-down) approaches that neglect contextual realities such as community history, current resources, provider experience and geographic realities perpetuate the tendency towards uniformity, usually to the disadvantage of heterogeneous rural communities. When the Rural Birth Index was developed, calculating the level of need (the ‘deterministic’ phase) was the first of three steps, the subsequent ones being the feasibility and prioritizing stages. The feasibility stage is guided by the question, “*What are the pragmatic issues that need to be considered in locating a particular health service in a given rural community?”* [1]. The literature abounds with studies that evaluate the efficacy and effectiveness of health interventions but relatively little research is focused on how to successfully implement or scale up health programs and initiatives [9]. The perspectives of front-line workers, especially perceived barriers and the demands that a change of practice would impose, are key considerations when planning to implement health system changes [9]. The perspectives of people who will be affected by the change might be different from a group of outside researchers or panel of experts. Individuals play a significant role in the outcomes of innovation and are not just passive recipients. For this reason, it is important to understand individuals’ knowledge and beliefs about the intervention or system change, their self-efficacy, or belief in their ability to implement goals, and their individual identification with the organization [10].

This paper explores the feasibility of applying the recommended maternity service delivery levels to rural communities across British Columbia, from the perspectives of health care providers who work in communities that had a different level of service from that suggested by the RBI. By clearly understanding the inhibitors to meeting population level need, system interventions can be developed and targeted to assist in achieving optimal maternity care for rural communities.

## Background

The RBI was first developed and applied in British Columbia in 2009 [1]. In 2019, BC’s Ministry of Health commissioned a refresh of the RBI to reflect changing health care and demographic circumstances. In the interval between the two calculations, the RBI was adapted to the Australian context (‘Australian Rural Birth Index’- ARBI) and applied nationally to inform the Australian National Maternity Services Plan [11]. Part of this work included the development of a toolkit to assist health planners in implementing the ARBI’s recommended services levels [12]. To this end, the ARBI toolkit encourages health system planners to consider several issues including demographic trends and “service networks, clinical governance and risk” which requires evaluation of the strength of a regional, networked approach to rural services based on established and productive relationships with regional referral centres. Reciprocally, the toolkit also recommends consideration of the community’s role in supporting regional care for residents that may drain into the community from outlying areas. This requires a risk assessment of the consequences of *not* providing local care. “Community consultation and service models” considers the role of service models that reflect the desires of the community while “transport logistics” focuses on patient flow and local availability of emergency transport. The toolkit also recommends a focus on “Workforce” issues and urges consideration of appropriate provider capacity and the potential for upskilling as well as recruitment and retention of staff, while “physical infrastructure and resources” requires sufficiency of the physical environment and any upgrades that may be required (i.e., an operating room if introducing surgical services). This comprehensive consideration of the feasibility of implementing a level of care responsive to population need is a helpful guide; understanding the issues, however, is an upstream step that will aid in higher level service planning. This study set out to consider why some communities did not have a level of care predicted by the RBI modeling. The specific objectives were to: (1) assess whether the predicted level of care (i.e., surgically supported care or care without local access to local caesarean section) was appropriate for communities and (2) to understand and document the barriers to achieving the predicted level of care from local administrators and care providers.

We have used a pragmatic definition of “rural”, based on *level of perinatal health services available in the community*, underscored by an assumption of health services corresponding to population level need. To this end, any community without local access to specialist obstetrical services (a proxy for communities larger than approximately 15,000) is included in our study definition.

## Methods

This research is part of a larger mixed methods study on the expansion and recalculation of the Rural Birth Index in British Columbia [1]. The purpose of the qualitative field work described in this paper was a) to validate the predicted level of service in those communities where service levels were not in alignment with the RBI predictions and b) understand feasibility issues of implementing the recommended level of service from health care providers and administrators engaged in local health care.

### Setting and participants

Qualitative field work was conducted at 10 rural hospital sites in British Columbia. The hospital sites were selected to include a mix of communities in which the RBI score had changed more than 10% over the last ten years, and was not currently aligned with the updated RBI predicted level of service. Health service administrators and maternity service practitioners (physicians, obstetricians, nurses, and midwives) were invited to participate in an interview or focus group. To recruit, we contacted health service administrators to distribute our invitation to participate to eligible maternity service practitioners in the community.

### Data collection and analysis

Interviews and focus groups were semi structured and recorded. Participants were given a consent form ahead of the interview and given the opportunity to ask questions. All participants were informed of their right to withdraw from the study at any time. Verbal informed consent to participate was obtained at the start of the interview, after clarification of the study objectives and activities. Ethical approval for the study was obtained from the Behavioural Research Ethics Board of the University of British Columbia (BREB H20-00,652) and the participating regional Health Authorities. The University of British Columbia Behavioural Research Ethics Board approves of recorded verbal informed consent. All study protocols were carried out in accordance with relevant guidelines and regulations as approved by the review board.

Interviews were conducted via Zoom, a videoconferencing platform, and ranged from 45 to 90 min (average, 60 min). Qualitative field work was completed until we reached conceptual saturation.

Interview transcripts were digitally recorded and transcribed verbatim. The transcripts were coded using thematic analysis, a method for systematically identifying patterns and themes across a data set [13]. Two members of the research team (JK & NC) generated a codebook to guide the coding process. Coding was an iterative process and was refined throughout in order to fully capture all of the participants’ views. In cases where there were discrepancies, themes were reviewed and merged with other suitable themes.

## Results

A total of 14 participants from 10 communities across the province took part in the study. Themes included the challenge of maintaining obstetrical skills in low volume communities (including impact on provider confidence and reduced opportunities for clinical coaching), challenges recruiting and retaining health care providers (including inadequate remuneration of maternity care and ineffective practise models and a reluctance of providers to offer local maternity care) and issues with high acuity patient transport. Each one is described in more detail below.

### Maintaining obstetrical skillsets

#### Low birth volumes and provider confidence

In communities where the level of care predicted by the RBI did not match the existing level of care, participants identified the inability to maintain their obstetrical skillsets as a barrier to implementing the appropriate level of maternity care. Participants expressed that local birth volumes were too low to maintain key obstetrical skills, as they “do not see enough cases to feel comfortable” with performing crucial procedures such as c-sections. Some participants explained that while they “love obstetrics, … it takes a lot of exposure to maintain competency, and [we] are not going to get that in [my community]”. One participant indicated that in their catchment, birth volumes were so low that “sometimes you can be delivering babies with nurses that have never seen a baby being born. That creates an unsafe birthing experience when none of our skills are up to par”. With low birth volumes and a lack of trained maternity staff, some participants feel that offering a full scope of maternity services is not feasible for their hospital, regardless of the community’s strong desire for local births. One participant explained,

*“[It is not] feasible to offer caesareans for six people a year. In the future, I agree that [lack of caesarean section] is certainly something that detracts from people coming here, but at this current point in time we're not there yet.”*

Even though some physicians with expanded surgical skills may possess the adequate maternity training to uphold local services, participants explained that having procedural skills without the ability to practice what was learned in training “is not quite useless, but not far from it”. An absence of opportunities to refine their maternity skills has left several participants lacking confidence in their skillsets, as one physician noted,

*“Just don’t get enough [deliveries] to feel comfortable ... . I just spent six months at the Women's and Children's Hospital and I am barely feeling comfortable after 100 deliveries in 6 months. When we are having 2 deliveries a year in [community], we're never going to develop that confidence.”*

This is especially true in rural community hospitals without robust maternity services, where frequent unplanned or emergency deliveries have occurred. In communities without maternity services, many rural birthers chose to travel to deliver their babies in centers with higher levels of care, while others were adamant about delivering locally for cultural, economic and personal reasons. One participant explained that due to the characteristics of the childbearing population in their community,

*“Most individuals would be early if they’re delivering here, all of them being under 37 weeks. They have not left the town or are delivering at 28 weeks- which is the scariest of all. It’s really hard to feel confident in deliveries when you do them so intermittently. Especially if you’re getting into neonatal resuscitation and those kinds of things, it’s always terrifying.”*

Preterm and emergency births can be “extraordinarily stressful” for maternity care providers, especially those who may not have surgical backup to rely on for support. Participants explained that a lack of surgical backup is a common characteristic of their local maternity services, where there is no surgeon available to provide emergency c-sections and other critical obstetrical procedures. Others expressed that their hospitals “have a low tolerance for accepting delivery with no c-section” backup, and several sites have “felt that they couldn't really provide maternity care anymore” following the loss of staff members holding c-section privileges. The absence of surgical backup combined with a lack of provider confidence due to low birth volumes continue to present as active barriers to achieving adequate local maternity services in rural communities.

#### Reduced clinical coaching opportunities

Another barrier impacting the feasibility of meeting the predicted level of maternity services is the lack of coaching opportunities available to local care providers. Maternity coaching opportunities often come with high monetary costs associated with hiring educators, registration costs, and travel expenses. Several participants have expressed that “there was no funding” to ease the financial burdens associated with enrolling multiple staff members in maternity training courses. One participant expressed that “the cost to send somebody [for training]- between the time away from home, travel, and costs to my organization, it’s $10,000 for one nurse to go through” a maternity training program. Others indicated that care providers must have a willingness to take time off to travel to larger urban centres, where the majority of obstetrical training is held. When asked about their experience with constant travel to urban centres to maintain their obstetrical skillset, one participant said,

*“I used to be someone who would have all of their OB skills up to date. I used to travel to Burnaby to do obstetrics there because I loved it so much until it became too much to handle. Up until two to three years ago I was fully certified with everything: fetal resuscitation, strip reading, everything, but I just let it go because it wasn't worth it here and I didn't want to travel anymore.”*

Even when sufficient funding is available to reimburse rural providers for travel and other coaching expenses, a lack of provider engagement and “less interest from staff towards participating in education” persists. The organization and implementation of maternity coaching sessions is a process that can span over several months, and in some instances hospital sites had “educated lots of people, but as time went on and the training process got dragged out, no one wanted to be educated anymore”. Some participants explained that “[support] staff rotate regularly”, and so organizing coaching sessions with high enrollment rates can be a “real challenge”. Despite the barriers to participating in maternity training opportunities, many participants still felt that increasing training initiatives for rural providers is essential to supporting the expansion of maternity services to meet community need.

### Recruitment and retention of care providers

#### Recruitment of maternity nurses and physicians

Rural hospital sites continue to face challenges with the recruitment and retention of physicians and nurses in their communities, exacerbated by the declining birth rates in some communities. Several participants indicated that due to the low volume of local deliveries and a frequent lack of surgical backup for maternity care, prospective health care providers were reluctant to practice in their community. Many participants also stated that nursing shortages have been a longstanding issue, and the amount of trained maternity nursing staff remains very low. Among those in practice, in some communities less than half have a current neonatal resuscitation program (NRP). Several participants explained that historically, their sites have had to suspend services and go on diversion due to a shortage of nursing staff, so it became difficult for sites to offer 24/7 access to maternity care. When asked about the stability of their nursing team, one participant explained,

*“Currently, [our community] has 5 out of 11 nursing positions occupied. It has a 50% vacancy in nursing positions, and there have been weeks were there has not been a single nurse put to work in that site. … It's a major, major blow. One of the things that I've seen since I started here is that we have been in this position with nursing before. That’s why we lost services in the first place in 2004, because I don't even think we had one nurse at one point. So it's pretty grim right now.”*

Rural sites not only face challenges with recruiting new maternity staff, but they also experience difficulty retaining existing practitioners. Participants reported “movement [of staff] in all sectors”, where sites experienced new midwives coming and old midwives leaving or retiring, with the same cycle occurring for physicians and obstetricians. Several participants explained that a key factor influencing physicians’ decisions to leave rural hospitals that offer maternity services is due to, as noted above, lack of confidence in their delivery skills combined with lack of interest in being on call for deliveries without adequate remuneration. The ongoing difficulties with physician recruitment and retention highlight the need for effective strategies to retain practitioners in the community to support the expansion of local maternity services.

#### Inadequate remuneration for maternity care

In order to increase recruitment and retention, participants explained that appropriate monetary compensation is critical to incentivize physician and nurse participation in maternity care on top of their current family practices and other roles at the hospital. In some communities, there has been minimal interest from current staff towards supporting maternity care due to a lack of financial incentive to expand their practice to include work that is not financially remunerated. Some participants agreed that “it has come down to the all-mighty buck. The liability insurance, and the fact that it's really time consuming. It is just not profitable for doctors to do obstetrics anymore”. Many rural care providers are on fee for service payment plans, which can be detrimental to care providers who attend so few births per year. When asked about the feasibility of participating in a maternity clinic on a fee for service model, one participant said:

*“Currently we are working fee for service, so taking a day of your practice to spend at a woman's clinic would not be profitable, even though I'd be willing to engage in it. I'm an older physician; I'm set in my finances, and I'm not as eager to get the financial reward of fee for service. But we have several physicians who are new in their practice that are fee for service that would not want to spend a day at a woman's clinic because it wouldn't be profitable for them. But we're hoping to change that, and I think within the next 6 months, we'll see contracted salaries for physicians, and we hope to attract more physicians here.”*

Several participants agreed that the fee-for service model remains one of the largest barriers to increasing physician engagement with local maternity care, and that alternative payment options must be considered to support a robust maternity care team and attract physicians and midwives to rural areas. Many participants explained that this would stabilize services in rural settings by allowing providers to commit to providing maternity services without worrying about the negative impact that low birth volumes would have on their income. As one participant said:

*“If [the Alternative Payment Plan contract] goes through, optimally we'd like to have three full-time positions at each clinic. And if that's the case, I think we would be able to facilitate a better woman's clinic and hopefully we'll have ultrasound services here. At that point in time, we might be able to talk about planned deliveries… . But at this current time, the feelings of the physicians are [that] it would be too risky to have planned deliveries… . So, I think that's where the physicians stand at this point.”*

Many participants agreed that halting the use of fee for service models and adopting APPs could remove the financial barriers preventing some rural communities from providing their RBI predicted levels of maternity care.

#### Provider attitudes towards local maternity services

Due to inadequate remuneration, a lack of surgical backup, and difficulties maintaining obstetrical skillsets in rural settings, several providers expressed no interest in supporting local maternity care. Several participants felt that if their hospitals started to offer planned deliveries, there would be an exodus of physicians from practice. At sites with existing maternity clinics, participants explained that working in the clinic is viewed as a choice, not a mandate, and that it was essential to allow for this flexibility. When asked about their attitude towards providing maternity care at their hospital, one participant said:

*“It is already hard enough to get nurses to work in a rural site. To add the factor of being a delivery site is another turn in the screw where nurses will say ‘hey, I don’t want to work here because you deliver babies.’ I’m guilty of that. I don’t want to deliver babies- I didn’t become a nurse to deliver babies. But I live here so I’m dealing with it.”*

Some participants argued that the ongoing struggles with recruitment, combined with negative provider attitudes towards maternity care make the expansion of maternity services to meet the RBI predicted level of care unfeasible. One participant explained that “with the way our services are now, I can't see us safely expanding to provide scheduled deliveries here”. This highlights the need to address the root causes of rural care provider’s negative perceptions of local maternity services in order to provide adequate obstetrical care close to home.

### Patient transport

An additional factor preventing these communities from achieving their RBI predicted level of maternity service is the reliability of local transport. Some participants were hesitant to expand maternity services in their community without dependable transport to facilitate emergency transfers of patients to higher levels of care when necessary. They explained that it can be “a scary area if a c-section is needed, as [the referral community] is two hours away from here” and “laboring patients are already challenging to send out from a transfer perspective”. Others expressed that relying on transport for patients experiencing serious birth complications can be “burdensome and too stressful for the nurses and physicians”, especially in the event that an expedient transfer is not available. Several participants highlighted their frustrations with the BC ambulance service and indicated that *“*even if we did want to start an intrapartum wing for c-section, we would need to have transfer that's reliable, and we don't at the moment. Not at the speed that you need for a c-section”. One participant recalled their experience with transporting a labouring patient,

*“I myself have gone up to [community] in an ambulance to bring a maternity patient back here in an ambulance that was close to delivering. She didn't deliver in the ambulance, but that was a possibility, and it put people in a very difficult situation. I had a patient here a while ago that had a fractured pelvis who was told that she could never deliver [vaginally]. It was in the middle of winter, so we did not have transportation, we didn't have c-section. I was just told I must just manage her and deal with it.”*

The extreme meteorological and geographical conditions characteristic of rural communities in BC, especially during winter, are one of the major barriers to timely patient transfers. When asked about how intermittent weather conditions impact the reliability of ambulance transfers for labouring patients in their community, one participant explained,

*“It depends on the weather and on the patient. I certainly don't think that a low acuity ambulance driving a patient in labour to [referral community] is ideal. For a helicopter or plane evacuation, we are dependent on the patient transfer network for these kinds of cases, but then [the service] is still dependent on the weather. So even if we had reliable air transportation, if it is in the middle of winter with snow then they can't fly.”*

Without local surgical capabilities and dependable services to transport patients to higher levels of care, many participants believed that the expansion of maternity care to meet community needs is unfeasible.

### Limitations

This study was undertaken in rural British Columbia with a select group of communities that do not have the appropriate level of service as per the RBI calculations. These reasons may not be transferable to other jurisdictions with other health service delivery arrangements or to other time periods when recruitment and retention of health care providers many not be as urgent.

## Discussion and conclusion

Participants articulated three thematic challenges to providing maternity services in their communities: maintaining clinical skills and financial stability in the context of low procedural volume, recruitment and retention of care providers and challenges with patient transport.

These findings align well with results of a review of rural hospital policies in eight high income countries, including Canada [14]. Financial sustainability, health professional shortages, timely transport to higher levels of care and the importance of medical education and telehealth were the main issues identified. Unlike some other jurisdictions, Canada does not have a national policy that outlines the role of rural hospitals and there is no law or policy specifying catchment characteristics or size “for the establishment or maintenance of a hospital.” ([14] p. 763).

In the face of the precipitous attrition of rural maternity services across BC, Canada and internationally in the past two decades, there has been a growing body of evidence identifying system vulnerabilities that must be addressed before services can be stabilized. They include but are not limited to issues that plague the larger health system, exacerbated by the COVID-19 pandemic, such as recruitment and retention of care providers [15–17] and inadequate remuneration for providing maternity care, particularly due to lack of financial acknowledgement of on-call commitments [18, 19]. Other issues that have been well documented specifically in rural settings include the challenge posed by the very nature of rural practice itself: low population densities, often over vast geographies. Although this leads to practical barriers such as reduced opportunities for clinical coaching and maintaining provider confidence, it does not reduce rural populations’ need for appropriate (local) care.

Evidence has also pointed to system interventions to mitigate the challenge of providing care in rural communities, ranging from alternative compensation models, additional clinical support for health care providers and building lateral networks of care provider support between rural communities and higher levels of care [20]. However, solutions are vulnerable to failure if we do not view current challenges through a *health systems* lens. That is, sustainability is no longer a concern of only low-volume rural sites, but instead afflicts services across the continuum of care with those ‘up-stream’ forced to contend with increased volume of maternity patients due to the outsourcing of smaller services in addition to the wide-spread recruitment, retention and compensation challenges. To avoid the deleterious outcomes of a ‘domino’ effect, we must move away from piece-meal solutions and prioritize our social responsibility to provide optimal care to birthing families. This starts with rethinking compensation models.

Participants in this study pointed out that in a fee-for-service environment, there is a lack of parity for funding maternity care relative to other clinical responsibilities, such as emergency medicine or hospitalist work. This disparity, on top of rising costs for liability insurance, can lead to ‘break-even’ scenarios for care providers offering maternity care, at best. This is exacerbated in a fee-for-service environment where compensation may be an inadequate marker of actual time spent caring for patients and, in rural settings, where the low volume of patients may not afford the desired remuneration. Several participants in this study pointed to the value of Alternative Payment Plans (APP), where a salary is provided to meet population need for care, regardless of volume. In this way, APPs can address the burden of low volume by removing the financial penalty of practice. It should be noted, however, that compensation is not only about remuneration: for many, it is a proxy for respect for the services provided which extends beyond compensation for the actual tasks performed to perceptions of the valuing on maternity care. For example, many maternity care providers, particularly in low-volume settings, are challenged by on-call responsibilities as they must arrange their lives accordingly (by being available and ready to provide care), but without direct compensation. This is particularly burdensome in low volume settings where there are fewer care providers to share the burden of call alongside the potential for more acute situations due to the lack of other local resources.

The importance of remuneration in supporting sustainable health system change and interprofessional collaboration have been documented by others [9, 21, 22]. For example, Wranik et al. used a combination of document analysis and interviews with 33 primary care decision makers across Canada, to create a conceptual framework for funding and renumeration of interdisciplinary primary care teams. They found that key stakeholders favored a model where ‘provider remuneration is interdependent and combined with a team funding model that is linked to whole team activities’ ([21] p. 9). Main barriers identified were the existence of multiple funding lines (with multiple lines of accountability), lack of a clear funding line for space and equipment and a financial hierarchy/financial barriers to interprofessional collaboration (e.g. when physicians get funded for team activities but not other members of the health care team) [21, 22].

A qualitative study with fourteen rural fee for service primary care physicians in Alberta showed that the main barriers to rural practice were: high work load, the burden of being on call, the time required to keep up a broad skill set, inadequate access to specialists, outdated equipment (which affected patient care) and unfavourable changes to the billing structure. Most physicians were willing to explore alternate payment models and saw APPs as a way to recruit and retain staff. Participants noted the importance of APP contracts that were developed in collaboration with physicians, were clear and included adequate compensation [23].

The Rural Birth Index is an important health human resource planning tool for rural areas of the province, and implementing it will require significant political will and transformational leadership [10, 24]. Frameworks such as PARIHS (Promoting Action on Research Implementation in Health Services) are valuable tools when implementing and evaluating changes in health care organization or practice [24]. This framework proposes that three core components are needed to successfully translate evidence to practice: 1) Clarity around the evidence being used and a recognition that implementation will be most successful when the evidence is scientifically sound and aligns with patient and provider preferences; 2) people with appropriate roles, skills and knowledge who can support teams and organizations in applying evidence, like the RBI, into practice. These champions need to be able to adapt to changing situations, support group processes, and promote critical thinking and 3) willingness of leaders to transform and shape culture, by promoting continuous learning, flexibility and attention to both the needs of individuals and the group. In other words, transformational leaders ‘are those that create contexts conducive to integration of evidence into practice’ ([24] p. 299).

The values propositions that must guide compensation discussions are simple: for rural communities with enough deliveries for providers to maintain competency over time, it is the social obligation of our health care system, within the context of commitment to equity, to provide local access to maternity care. Metrics like the Rural Birth Index can point to where such volume-distance-vulnerability thresholds lie. Once we understand this as a matter of ethics and responsibility instead of economies of scale and striving for economic neutrality (maintaining and managing health system costs), we can truly work towards solutions. It is essential, however, to remove volume thresholds from such practice arrangements once it has been decided that local access to care is a health system priority. It is likely that other rurally-specific challenges noted by participants in this study and reported in the literature will be mitigated once compensation for low volume settings is addressed.

## Data Availability

The datasets generated and analyzed in this study are not publicly available to prevent participant identification, due to the small number of participants in a localized area but are available from the corresponding author on reasonable request.

## Abbreviations

RBI: Rural Birth Index
ARBI: Australian Rural Birth Index
NRP: Neonatal Resuscitation Program
APP: Alternative Payment Plans
PARIHS: Promoting Action on Research Implementation in Health Services
